# unsurv: clustering individualized survival curves

**DOI:** 10.1093/bioadv/vbag218

**Published:** 2026-08-06

**Authors:** Imad El Badisy

**Affiliations:** AI and Data Science Service, Mohammed VI Center for Research and Innovation (CM6RI), Rabat, Morocco; Mohammed VI University of Sciences and Health (UM6SS), Casablanca, Morocco

## Abstract

**Motivation:**

Biomedical phenotyping workflows often cluster high-dimensional molecular or clinical covariates and then test survival differences post hoc. This can miss structure that is expressed primarily through the timing of risk, because two patients may have similar scalar risk at one horizon but different predicted survival trajectories. Many survival learners can output patient-specific curves ${\left\{{\hat{S}}_{i}(t)\right\}}_{i=1}^{n}$ on a common time scale. Clustering these curves gives a survival-native post-prediction representation for exploratory phenotyping. Existing work on survival-curve clustering mainly targets predefined Kaplan-Meier curves or general functional data, whereas a practical tool for individual-level predicted survival curves remains less developed.

**Results:**

We introduce unsurv, an R package that clusters individualized survival curves on a common time grid using validity-preserving preprocessing, time-weighted integral distances, and partitioning around medoids (PAM). The output includes observed medoid prototype curves, a nearest-medoid rule for assigning new patients, model-selection summaries, resampling-based stability using the Adjusted Rand Index (ARI), and a new unsurv_compare() function for comparing partitions against baselines. The package is model-agnostic and can be paired with any survival learner that returns ${\hat{S}}_{i}(t)$. We illustrate the workflow on a synthetic example and on the METABRIC breast cancer cohort using survdnn-predicted survival curves, including baseline comparisons and an out-of-sample validation check showing that the curve-based cluster ordering generalizes to held-out patients.

**Availability and implementation:**

The unsurv package is available on CRAN and its full source code and documentation are provided at https://github.com/ielbadisy/unsurv under the MIT License.

## 1 Introduction

### 1.1 Background and motivation

Unsupervised phenotyping is widely used in computational biology and translational oncology, especially for multi-omics subtype discovery. A typical workflow clusters molecular features (or learned embeddings) and then tests whether the resulting groups differ in survival (e.g. Cai and Wang 2024, Yang *et al.* 2025). This workflow is convenient, but the object optimized by the clustering algorithm is usually a covariate representation rather than the time-to-event prediction itself. Consequently, clusters can be stable in the covariate space while still failing to separate patients by when risk accumulates over follow-up.

Predicted survival curves provide a different object to cluster. Many survival learners, including penalized Cox models, random survival forests, and deep survival networks, can output individualized survival trajectories ${\hat{S}}_{i}(t)$ rather than only a scalar risk score. These trajectories encode *when* risk accumulates. Two patients with similar risk at a fixed horizon can still show different curve shapes (early decline, delayed separation, plateauing), which is often the aspect clinicians and translational scientists care about.

Most methods that “cluster survival curves” address a different problem: grouping *predefined* Kaplan-Meier curves across known groups. These approaches often embed hypothesis-testing logic and select the number of groups using resampling or log-rank-based criteria (Villanueva *et al.* 2019, Villanueva *et al.* 2025). Functional clustering methods provide a broader toolkit for curve-valued data, including basis-expansion and functional principal-component approaches (Abraham *et al.* 2003, Jacques and Preda 2014). Functional survival comparison procedures also emphasize localized deviations over the follow-up window (Grollemund *et al.* 2024). What is less available is a simple, deployable post-prediction layer that works directly with *individual-level predicted survival curves* produced by an arbitrary chosen learner.

We present unsurv, an R package for post-prediction phenotyping based on clustering individualized survival trajectories using time-weighted integral distances and partitioning around medoids (PAM). Although it uses established clustering components, the package defines a survival-native workflow: (i) work directly with ${\hat{S}}_{i}(t)$ rather than covariates or scalar risk scores, (ii) project curves to a valid bounded and non-increasing survival-function space, (iii) represent similarity through integrated L1/L2 distances over time, (iv) use observed medoid curves as interpretable temporal-risk prototypes, (v) assign new patients by nearest medoid after the same preprocessing, and (vi) report stability under resampling.

### 1.2 Statement of need

High-dimensional molecular and clinical data are increasingly used to train survival models. In this setting, the high-dimensional problem is handled upstream by the chosen survival learner, while unsurv operates downstream on the resulting low-dimensional functional object: the predicted survival curve sampled over time. If the scientific question is about temporal risk profiles, clustering in the survival-curve domain is a natural complement to clustering the original covariates.

unsurv addresses this need by clustering predicted survival trajectories. Phenotypes are defined by temporal risk profiles rather than by patterns in the original covariates. This is complementary to subtyping pipelines that learn embeddings for clustering and then evaluate survival separation (e.g. Cai and Wang 2024, Yang *et al.* 2025), and it differs from methods designed to group predefined Kaplan-Meier curves using bootstrap or log-rank-based procedures (Villanueva *et al.* 2019, Villanueva *et al.* 2025). The METABRIC breast cancer cohort, which combines molecular measurements with long-term clinical follow-up (Curtis *et al.* 2012, Pereira *et al.* 2016), provides a representative use case for such a post-prediction phenotyping layer.

### 1.3 Contribution

This application note provides:

1. A post-prediction phenotyping approach based on individualized survival trajectories ${\hat{S}}_{i}(t)$.
2. Time-weighted integral distances (L1/L2) implemented via normalized trapezoidal weighting on a common time grid.
3. Medoid-based clustering (PAM) with interpretable prototype curves.
4. Out-of-sample assignment for new patients via nearest medoid.
5. Resampling-based stability assessment using the Adjusted Rand Index (ARI).

## 2 Methods

### 2.1 Input representation

Let *n* denote the number of individuals and $t_{1}<\ldots <t_{Q}$ a strictly increasing time grid. For each individual *i*, we observe or predict a survival curve sampled on this grid:


$$S_{i}=(S_{i}(t_{1}),\ldots ,S_{i}(t_{Q})),\;i=1,\ldots ,n.$$

The admissible curve space is


$$S_{Q}=\{u\in {\left[0,1\right]}^{Q}:u_{1}\geq u_{2}\geq \cdots \geq u_{Q}\}.$$

Stacking rows yields a matrix $S\in {\left[0,1\right]}^{n\times Q}$. The goal is to partition the *n* trajectories into *K* clusters based on similarity of their temporal survival behavior. In practice, *S* is typically a matrix of predicted curves ${\hat{S}}_{i}(t)$ from a selected survival learner.

### 2.2 Preprocessing

Survival probabilities should remain within [0,1] and should be non-increasing in  t.  unsurv therefore applies a deterministic validity map $\Pi :R^{Q}\to S_{Q}$ before computing distances.

**Clamping**


$$S_{i}(t_{q})\leftarrow \text{min}\{1,\text{max}\{0,S_{i}(t_{q})\}\}.$$

**Monotonicity enforcement**


$$S_{i}(t_{q})\leftarrow \text{min}(S_{i}(t_{q}),S_{i}(t_{q-1})),\;q=2,\ldots ,Q.$$

**Optional median smoothing** (odd window width w≥3)


$$S_{i}(t_{q})\leftarrow \mathrm{median}\{S_{i}(t_{r}):r\in [q-h,q+h]\},\;h=(w-1)/2.$$

These steps are particularly useful when curves come from flexible learners and may show small local irregularities. Related curve-centric pipelines also apply monotonicity-preserving interpolation and smoothing to stabilize downstream comparisons (Grollemund *et al.* 2024).

### 2.3 Time-weighted integral distances

Distances are defined to approximate normalized continuous-time integrals over the follow-up window. Let $\Delta_{q}=t_{q+1}-t_{q}$. Define trapezoidal quadrature weights:


$$w_{1}=\frac{1}{2}\Delta_{1},\;w_{q}=\frac{1}{2}(\Delta_{q-1}+\Delta_{q})\;(q=2,\ldots ,Q-1),\;w_{Q}=\frac{1}{2}\Delta_{Q-1},$$

and normalize them so that $\sum_{q=1}^{Q}w_{q}=1$.

### 2.4 L2 (integrated squared deviation)

We map curves into a weighted Euclidean space by


$$x_{iq}=\sqrt{w_{q}}S_{i}(t_{q}).$$

Then


$$\|x_{i}-x_{j}{\|}_{2}^{2}=\sum_{q=1}^{Q}w_{q}{\left(S_{i}(t_{q})-S_{j}(t_{q})\right)}^{2}\approx \frac{1}{t_{Q}-t_{1}}\int_{t_{1}}^{t_{Q}}(S_{i}(t)-S_{j}(t){)}^{2}dt.$$

### 2.5 L1 (integrated absolute deviation)

For L1 distances, we use


$$x_{iq}=w_{q}S_{i}(t_{q}),$$

so that


$$\|x_{i}-x_{j}{\|}_{1}=\sum_{q=1}^{Q}w_{q}|S_{i}(t_{q})-S_{j}(t_{q})|\approx \frac{1}{t_{Q}-t_{1}}\int_{t_{1}}^{t_{Q}}|S_{i}(t)-S_{j}(t)|dt.$$

Optional column standardization can be applied after weighting when early and late time regions are on very different scales, which can happen in long follow-up studies.

### 2.6 PAM clustering and model selection

Given the feature matrix *X*, we compute a dissimilarity matrix (Euclidean for L2; Manhattan for L1) and apply partitioning around medoids (PAM) (Kaufman and Rousseeuw 1990). For medoid indices $M=\{m_{1},\ldots ,m_{K}\}$, the fitted partition minimizes


$$\sum_{i=1}^{n}\underset{m\in M}{\min}d(x_{i},x_{m}).$$

PAM is used deliberately because each prototype is an observed valid survival curve, the method accepts arbitrary dissimilarities, and the fitted medoids give a direct assignment rule for future predicted curves. This differs from centroid-based functional clustering, where the cluster center may not correspond to an observed patient trajectory.

If *K* is not specified, unsurv scans $K\in \{2,\ldots ,K_{\text{max}}\}$ and reports the value maximizing the mean silhouette width (Rousseeuw 1987). The silhouette compares within-cluster cohesion with nearest-neighbor-cluster separation; in unsurv it is a pragmatic exploratory default rather than an inferential guarantee. Users can instead specify *K* from prior biological knowledge or inspect stability across candidate values. This differs from approaches that select the number of groups among Kaplan-Meier curves using hypothesis testing or resampling strategies (Villanueva *et al.* 2019, 2025).

**Algorithm 1** unsurv fitting **Require:** Survival matrix *S*, time grid $\left(t_{q}\right)$, preprocessing options, distance type, and either *K* or $K_{\text{max}}$

**Ensure:** Cluster labels, medoid indices, medoid curves, selected *K* (if applicable), and silhouette summary

 1: Apply clamping; optionally enforce monotonicity and smoothing

 2: Compute trapezoidal weights (or accept user weights)

 3: Construct the weighted feature matrix under L1 or L2 mapping

 4: Compute pairwise dissimilarities

 5: **if** *K* is unspecified **then** 

 6:  Select *K* by silhouette scanning

 7: **end if** 

 8: Fit PAM and return labels and medoids

### 2.7 Prediction for new patients

For a new curve $S_{\text{new}}$ on the same time grid, unsurv applies the same validity map and feature embedding Φ used during training, and assigns the nearest medoid:


$$\hat{c}(S_{\text{new}})=\text{arg}\underset{k}{\min}d(\Phi (\Pi (S_{\text{new}})),\Phi (\Pi (S_{m_{k}}))),$$

where $S_{m_{k}}$ denotes the stored medoid curve for cluster *k*.

### 2.8 Stability assessment

Because predicted curves inherit uncertainty from the upstream survival learner, unsurv reports stability under resampling. Subsampling without replacement and bootstrap resampling with replacement are supported. Optional perturbations include small additive noise on curves (followed by monotonicity enforcement), perturbation of time weights, and feature-space jitter.

Stability is summarized by the ARI (Hubert and Arabie 1985) between resampled partitions on overlapping indices. This focus on robustness is consistent with concerns raised in high-dimensional clustering and has motivated representation-learning approaches that aim to stabilize embedding-based subtyping (e.g. Cai and Wang 2024, Yang *et al.* 2025), although those methods operate on covariates rather than on survival trajectories.

### 2.9 Implementation

unsurv is implemented in R and uses cluster::pam for medoid-based clustering (Kaufman and Rousseeuw 1990). The main user-facing entry point is unsurv(), which takes a matrix of individualized survival probabilities evaluated on a common time grid and returns an “unsurv” object containing cluster assignments, medoid indices, medoid curves, preprocessing settings, and model-selection summaries when automatic selection of *K* is used.

The package API follows a standard R modeling workflow. New curves can be assigned with the S3 method predict() for fitted “unsurv” objects. Resampling-based robustness assessment is provided by unsurv_stability(), which repeatedly refits the clustering and summarizes agreement with the ARI. For visualization, the package provides plot() and autoplot() methods for fitted objects, together with plot_stability() for stability summaries. This revision adds unsurv_compare(), which takes a named list of cluster-label partitions on the same individuals (e.g. an unsurv curve-based partition together with one or more baseline partitions) and reports cluster sizes, ARI agreement with a reference partition, and per-cluster Kaplan-Meier medians, with a corresponding autoplot() method that plots Kaplan-Meier curves faceted by method; this is the functionality used to produce the baseline comparison in Section 3.2.

In practice, the intended workflow is simple: obtain individualized survival curves from any upstream survival learner, pass those curves and their time grid to unsurv(), inspect the returned medoid prototypes and cluster assignments, optionally use predict() for new patients, use unsurv_stability() when a robustness check is needed, and use unsurv_compare() when comparing the resulting partition against alternative clusterings.

Additional methodological details and the R script used to reproduce the main manuscript analyses are provided in the Supplementary Material.

## 3 Examples

### 3.1 Synthetic illustration

We first illustrate the workflow on a synthetic data-generating process where three latent risk regimes induce distinct survival-curve shapes. We simulated n=270 patients (90 per regime) from cluster-specific Weibull distributions with increasing shape and scale parameters across groups, then generated four covariates whose distributions also differed by latent class. Individual Weibull parameters were further perturbed by the covariates, so the latent structure remained recoverable but not perfectly separable from the observed features alone. We split the data into 189 training and 81 test patients, fit the survdnn package (EL-Badisy 2026) on the training set using the observed (time,status) outcomes, and predicted individualized survival curves for the held-out test set on a 100-point common time grid.

**Figure 1:**
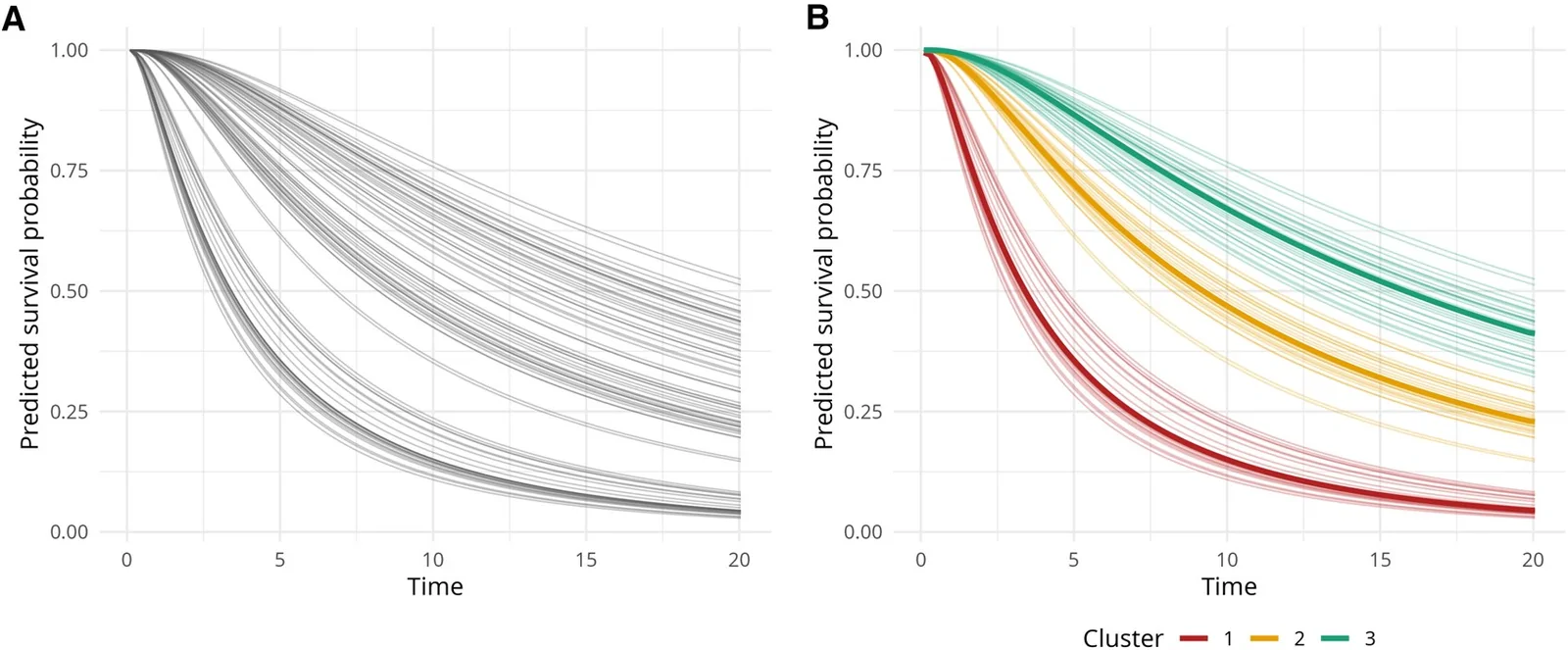
Synthetic data-generating-process example: (A) survdnn-predicted survival curves on the held-out test set and (B) the corresponding unsurv clustering with medoid prototypes.

We then applied unsurv to the test-set predicted curves using L2 distance, monotonicity enforcement, and K=3. Figure 1 shows the resulting structure and is generated in two steps: panel A plots the raw survdnn output (the predicted curves passed as the S argument to unsurv(), before clustering), and panel B is produced by calling unsurv() on those curves and passing the fitted object to the package’s plot()/autoplot() method, which colors each curve by its assigned cluster and overlays the medoid prototypes stored in fit$medoids. The comparison is informative because it shows what the partition adds: panel A alone gives no indication of temporal-risk subgroups, while panel B recovers three visually and numerically distinct trajectory groups directly from the predicted curves, without using the observed test-set outcomes. Recovery against the known latent regimes was nearly exact. The recovered cluster sizes were 26, 26, and 29, compared with true test-set sizes of 26, 27, and 28. The contingency table showed a single mismatch (one class-2 patient assigned to class 3), yielding a label-matched accuracy of 0.988 and an ARI of 0.962. This example illustrates the intended use case of unsurv: an upstream survival learner maps covariates to individualized trajectories, and unsurv converts those trajectories into interpretable temporal phenotypes.

### 3.2 METABRIC breast cancer application

We next applied the workflow to the METABRIC breast cancer cohort (Curtis *et al.* 2012, Pereira *et al.* 2016), using the version distributed in the biostatlab R package. After complete-case filtering, the analysis included 1839 patients with overall-survival time, event status, and 120 high-variance numeric clinical/molecular predictors.

Following a reviewer suggestion, we used a three-way split rather than a single train/test split, so that cluster generalization to unseen patients can be assessed directly. Patients were split into a training set (1103 patients, used only to fit survdnn), a partition set (367 patients, used only to fit unsurv and two baseline partitions), and a validation set (369 patients) held out from both survdnn fitting and partitioning. Survival curves were predicted for the partition and validation sets on a 100-point monthly time grid up to the 90th percentile of training follow-up.

unsurv with K=3 was fit on the partition-set curves, giving clusters of sizes 102, 144, and 121. We compared this with two baselines fit on the same partition set: PAM on a scalar predicted-risk summary at the median time grid point, and PAM on the first five principal components of the original predictors. Validation-set patients were then assigned to clusters out-of-sample: unsurv curves via predict() (nearest medoid), the scalar-risk baseline via nearest cluster-mean risk, and the covariate-PCA baseline via nearest cluster-mean centroid in PCA-score space (with PCA loadings fixed from the training set). Clusters were relabeled by partition-set Kaplan-Meier median survival (label 1 lowest, label 3 highest) so that labels carry the same meaning across sets and methods. Partitions were compared using the new unsurv_compare() function (unsurv package, added in this revision), which reports cluster sizes, ARI agreement with the unsurv partition, and per-cluster Kaplan-Meier medians; a log-rank test is deliberately not use, since testing a partition against the labels it was fit to separate is circular.

**Figure 2:**
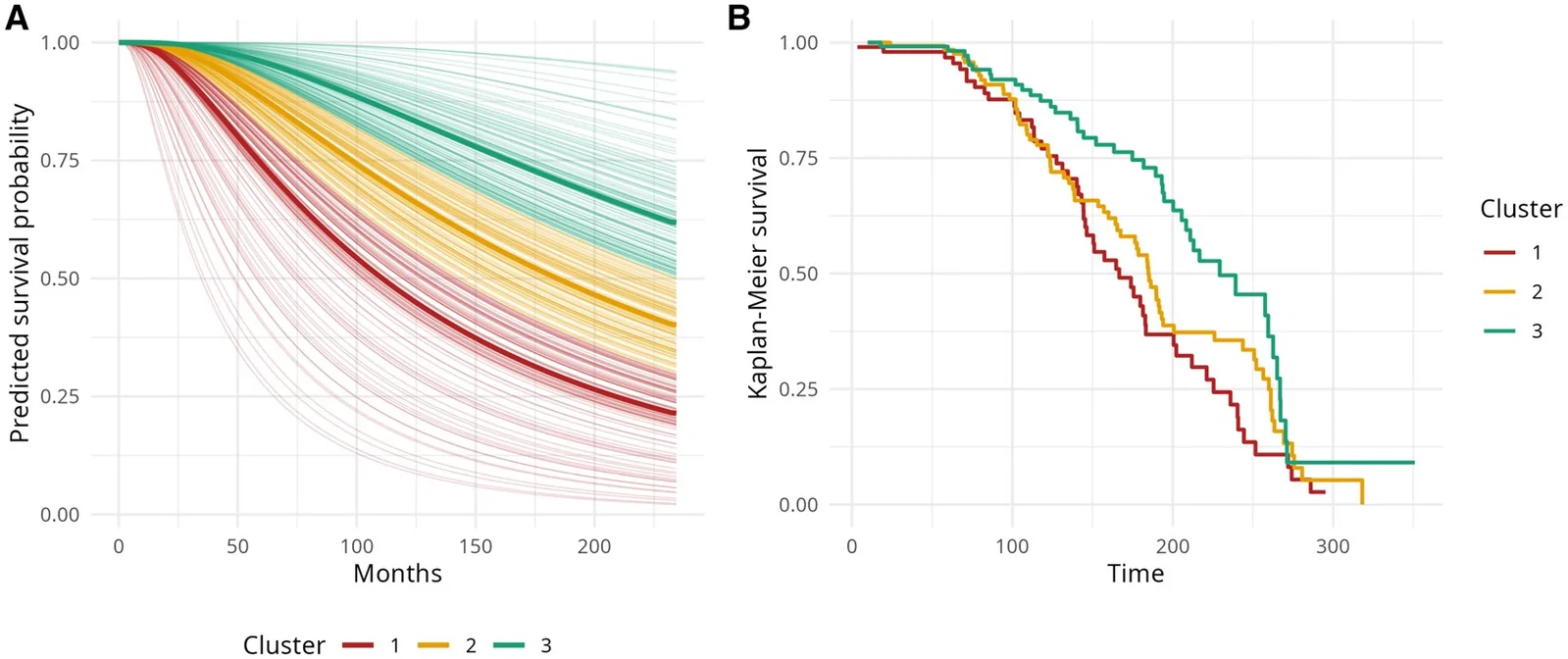
METABRIC application: survdnn-predicted survival curves clustered by unsurv on the partition set (left) and Kaplan-Meier curves for the resulting clusters (right). Panel A is produced by unsurv() followed by the package’s curve plot; panel B is a standard Kaplan-Meier plot of observed outcomes by cluster label, included to show that the curve-based partition, fit without using observed outcomes, recovers groups with distinct observed survival.

**Figure 3:**
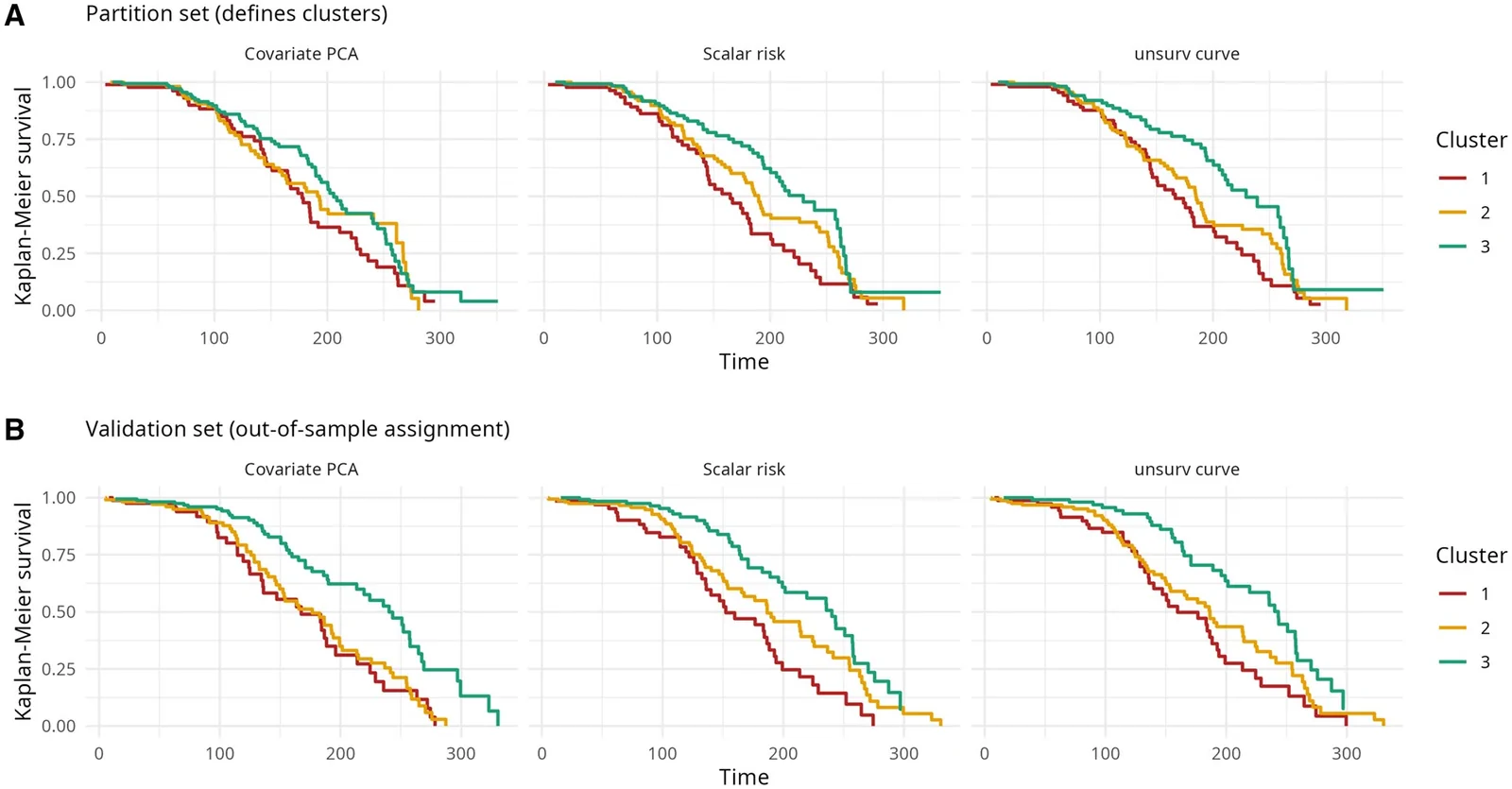
METABRIC baseline comparison and out-of-sample generalization, produced by unsurv::unsurv_compare() and its autoplot() method. Each panel shows Kaplan-Meier curves by cluster label for one partitioning method (unsurv curve clustering, scalar-risk PAM, covariate-PCA PAM). Row A uses the partition set on which each method’s clusters were defined; row B uses the independent validation set, with cluster membership assigned out-of-sample. Consistent label ordering (cluster 3 above cluster 2 above cluster 1) between rows A and B indicates that a method’s survival separation generalizes to unseen patients rather than reflecting overfitting to the partition set.

Table 1 reports the ARI against the unsurv partition and the per-cluster Kaplan-Meier medians for all three methods on both sets. For the curve-based partition, median survival increased monotonically with cluster label on both the partition set and the independent validation set, so the ordering “cluster 3 has better survival than clusters 1 and 2” generalized to unseen patients. Resampling stability on the partition set was good (mean ARI =0.849 over 30 subsamples) (Fig. 2). The scalar-risk baseline was highly concordant with the curve-based partition (ARI ≥0.81 on both sets) and also preserved its survival ordering out of sample, indicating that at this single horizon the scalar summary already captures much of the ordering information present in the full curve for this model. The covariate-PCA baseline was nearly unrelated to the curve-based partition (ARI ≤0.09 on both sets): it clusters a genuinely different representation, showed weaker partition-set separation between its two lowest clusters (median survival gap of 28 months, vs. 63–65 months for the curve and scalar-risk partitions), and only recovered a comparable gap on the validation set. Fig. 3 shows the Kaplan-Meier curves for all three methods on both sets.

**Table 1:** METABRIC partition comparison (K=3 for all methods).^a^

	ARI vs. unsurv		Cluster 1 median		Cluster 2 median		Cluster 3 median	
Method	Partition	Validation	Partition	Validation	Partition	Validation	Partition	Validation
unsurv curve	1.000	1.000	166.7	159.7	184.8	186.5	229.3	243.2
Scalar risk	0.833	0.810	164.7	152.3	189.7	186.6	229.3	240.2
Covariate PCA	0.018	0.086	177.6	167.4	193.1	176.6	205.6	240.2

a ARI is the Adjusted Rand Index against the unsurv curve-based partition, computed separately on the partition set and on the independent validation set. Cluster medians are Kaplan-Meier median survival (months); label 1 is the partition-set worst-survival cluster and label 3 the best, fixed by the partition-set ranking and applied consistently to the validation set. Produced by unsurv::unsurv_compare().

## 4 Discussion

High-dimensional clustering is often sensitive to noise and analytic choices, and it does not guarantee preservation of time-to-event structure. unsurv takes a different route by clustering individualized predicted survival curves. The resulting phenotypes are defined by temporal risk profiles, and the medoid representation makes the output easy to communicate.

The closest methodological neighbors are approaches that group Kaplan-Meier curves across predefined groups using bootstrap-based or log-rank-based rules to determine the number of groups (Villanueva *et al.* 2019, Villanueva *et al.* 2025). unsurv addresses a different setting: clustering at the individual curve level with out-of-sample assignment and explicit resampling-based stability. Functional clustering methods provide a broad foundation for curve-valued data (Abraham *et al.* 2003, Jacques and Preda 2014); unsurv is narrower by design, focusing on bounded monotone survival trajectories, observed medoid prototypes, and direct integration with predicted survival curves. Finally, while multi-omics subtyping methods cluster learned embeddings and assess survival separation post hoc (Cai and Wang 2024, Yang *et al.* 2025), unsurv directly clusters the endpoint representation produced by the survival learner.

The main limitations are the quadratic memory cost of pairwise dissimilarities, the requirement of a common time grid, and the exploratory nature of the selected clusters. PAM is appropriate for moderate sample sizes where interpretable medoid prototypes are useful, but larger cohorts may require scalable medoid approximations or alternative clustering backends. A further limitation concerns the upstream learner: under a proportional-hazards model, every predicted curve is a scalar power transform of a common baseline survival function, so the L1/L2 curve-shape distances used by unsurv reduce to a monotone function of the scalar risk score, which trivializes the curve-clustering step. unsurv is therefore best suited to survival learners that do not impose proportional hazards, such as random survival forests or flexible neural models like survdnn, where curve shape carries information beyond a single scalar summary; the METABRIC application in this note uses such a model. Extensions to competing risks and multistate predictions are natural directions.

## 5 Conclusion

unsurv provides post-prediction phenotyping by clustering individualized survival trajectories with time-weighted integral distances and medoid-based partitioning. It produces interpretable prototype curves, supports new-patient assignment, and reports resampling-based stability where high-dimensional clustering could be unstable.

## Supplementary Material

vbag218_Supplementary_Data

## Data Availability

The METABRIC data used in this study are publicly available from the original METABRIC studies. The unsurv R package, source code, and documentation are publicly available on CRAN and GitHub at https://github.com/ielbadisy/unsurv.
