# Supplementary material for: unsurv: clustering individualized survival curves
Source: Bioinform Adv. 2026 Aug 6;6(1):vbag218. doi: 10.1093/bioadv/vbag218 (PMC13524423; doi:10.1093/bioadv/vbag218)
Supplement: Supplementary file 1 [file vbag218_supplementary_data.pdf]

Supplementary Appendix

Additional details for the unsurv application note

Imad EL BADISY

1 Overview

This appendix provides compact descriptions of the synthetic example and METABRIC application used in the manuscript. The full R scripts used to generate the figures and summary files are included with the revised submission.

2 Synthetic example used in the main figure

The main figure illustrates the intended use case of `unsurv`: predicted individual survival curves are first obtained from an upstream survival learner, then clustered into temporal phenotypes.

We simulated three latent risk regimes with 90 patients per regime. Event times were generated from cluster-specific Weibull distributions with progressively larger shape and scale parameters across groups. Four observed covariates were also generated with class-dependent distributions, and these covariates slightly perturbed the individual Weibull parameters so that the latent classes remained recoverable but were not perfectly separable from the covariates alone.

The data were split into 70% training and 30% test sets. A `survdnn` model was fit on the training data with formula `Surv(time, status) ~ x1 + x2 + x3 + x4`. Predicted survival curves were then obtained for held-out test patients on a common 100-point time grid extending to the 90th percentile of the training follow-up time.

Clustering of predicted curves was performed with `unsurv` using:

- `K = 3`
- `distance = "L2"`
- `enforce_monotone = TRUE`
- `smooth_median_width = 3`
- `standardize_cols = FALSE`
- `eps_jitter = 0.0`
- `seed = 123`

To make cluster labels stable for reporting, the fitted medoids were ordered by their row means, which acts as a simple summary of average survival level across the time grid. The reported contingency table, ordered-label accuracy, and adjusted Rand index were computed on the held-out test set.

3 METABRIC application

The real-data application uses the `metabric` dataset distributed with the `biostatlab` R package. The analysis script is provided as `scripts/metabric_survdnn_unsurv.R`. It performs the following steps:

- loads METABRIC clinical, molecular, and survival variables;
- selects high-variance numeric predictors after complete-case filtering;
- splits patients into three disjoint sets: a training set (fits `survdnn`), a partition set (fits `unsurv` and the two baseline partitions), and a validation set held out from both survdnn fitting and partitioning;
- predicts individualized survival curves for the partition and validation sets;
- clusters the partition-set predicted curves with `unsurv`, and fits the scalar-risk and covariate-PCA baseline partitions on the same set;
- assigns validation-set patients to clusters out-of-sample: `unsurv` curves via `predict.unsurv()` (nearest medoid), the scalar-risk baseline via nearest cluster-mean risk, and the covariate-PCA baseline via nearest cluster-mean centroid in PCA-score space (with PCA loadings and centering/scaling fixed from the training set);
- relabels clusters within each method so that cluster labels reflect the partition-set Kaplan-Meier survival ranking (label 1 = worst median survival, label K = best), so labels carry the same meaning across sets and methods;
- compares the three partitions on the partition set and, separately, on the held-out validation set, using `unsurv::unsurv_compare()`, which reports cluster sizes, Adjusted Rand Index agreement with the `unsurv` partition, and per-cluster Kaplan-Meier medians (a log-rank test is deliberately not used, because testing a partition against the same labels it was fit to separate is circular);
- writes `results/metabric_summary.csv`, `results/metabric_cluster_summary.csv`, `results/metabric_unsurv_survdnn.png`, and `results/metabric_comparison.png`.

The manuscript reports the full run using 1839 patients after filtering, 120 predictors, 1103 training patients, 367 partition-set patients, and 369 held-out validation patients. If the survival ordering across clusters found on the partition set (e.g., "cluster 3 has better survival than clusters 1 and 2") is preserved on the validation set for a given method, that method's clustering is taken as generalizing to unseen patients rather than overfitting incidental structure in the partition set.

4 Reproducibility note

The code listing below is the exact synthetic-example code used to generate Figure 1. It is shown without execution output to keep the appendix readable. The METABRIC script is provided as a separate `.R` file because it is longer and writes multiple output files.

5 R script for the manuscript figure

```r
# ==================================================================
# Synthetic DGP -> train survdnn -> cluster with unsurv
# Three latent risk regimes are generated, survdnn learns them from
# covariates, and unsurv recovers the groups from predicted curves.
# ==================================================================

library(survival)
library(ggplot2)
library(survdnn)
library(unsurv)
library(patchwork)

set.seed(123)


# helpers
comb2 <- function(x) {
  ifelse(x < 2, 0, x * (x - 1) / 2)
  }

adjusted_rand_index <- function(labels_true, labels_pred) {
  tab <- table(labels_true, labels_pred)
  n <- sum(tab)
  sum_ij <- sum(comb2(tab))
  sum_i <- sum(comb2(rowSums(tab)))
  sum_j <- sum(comb2(colSums(tab)))
  expected <- sum_i * sum_j / comb2(n)
  max_index <- 0.5 * (sum_i + sum_j)
  if (max_index == expected) return(1)
  (sum_ij - expected) / (max_index - expected)
  }

extract_survmat <- function(pred) {
  if (is.matrix(pred)) return(pred)
  if (is.data.frame(pred)) return(as.matrix(pred))
  if (is.list(pred) && !is.null(pred$survival)) return(pred$survival)
  stop("predict() must return a matrix, data.frame, or a list containing $survival.")
  }

simulate_weibull_time <- function(shape, scale) {
  scale * (-log(stats::runif(length(shape))))^(1 / shape)
  }

# 1) Synthetic DGP with 3 latent classes
```

```r
n_per_cluster <- 90
cluster_specs <- data.frame(
  true_cluster = 1:3,
  shape = c(1.08, 1.35, 1.70),
  scale = c(6.0, 11.5, 19.5),
  x1_mean = c(-2.4, 0.0, 2.4),
  x2_mean = c(-1.6, 0.8, 2.8),
  x3_prob = c(0.20, 0.50, 0.80),
  x4_mean = c(2.0, 0.3, -1.2)
  )

sim_list <- vector("list", nrow(cluster_specs))

for (i in seq_len(nrow(cluster_specs))) {
  spec <- cluster_specs[i, ]

  x1 <- stats::rnorm(n_per_cluster, mean = spec$x1_mean, sd = 0.55)
  x2 <- stats::rnorm(n_per_cluster, mean = spec$x2_mean, sd = 0.60)
  x3 <- stats::rbinom(n_per_cluster, size = 1, prob = spec$x3_prob)
  x4 <- stats::rnorm(n_per_cluster, mean = spec$x4_mean, sd = 0.50)

  shape_i <- pmax(0.8, stats::rnorm(
    n_per_cluster,
    mean = spec$shape + 0.03 * x3 - 0.02 * x4,
    sd = 0.07
  ))

  scale_linpred <- 0.20 * x1 + 0.18 * x2 + 0.35 * x3 - 0.12 * x4
  scale_i <- spec$scale * exp(0.10 * scale_linpred + stats::rnorm(n_per_cluster, 0, 0.06))

  event_time <- simulate_weibull_time(shape = shape_i, scale = scale_i)
  censor_time <- stats::runif(n_per_cluster, min = 10, max = 28)
  obs_time <- pmin(event_time, censor_time)
  status <- as.integer(event_time <= censor_time)

  sim_list[[i]] <- data.frame(
    true_cluster = spec$true_cluster,
    time = obs_time,
    status = status,
    x1 = x1,
    x2 = x2,
    x3 = x3,
    x4 = x4
  )
  }

dat <- do.call(rbind, sim_list)
```

```r
dat$row_id <- seq_len(nrow(dat))

# 2) Train/test split
n <- nrow(dat)
idx_train <- sample.int(n, floor(0.70 * n))
train <- dat[idx_train, , drop = FALSE]
test <- dat[-idx_train, , drop = FALSE]


# 3) Train survdnn on observed times/status
fml <- Surv(time, status) ~ x1 + x2 + x3 + x4

mod <- survdnn(
  formula = fml,
  data = train,
  hidden = c(24, 12),
  activation = "relu",
  dropout = 0.0,
  batch_norm = FALSE,
  epochs = 160,
  lr = 5e-4,
  loss = "aft",
  optimizer = "adamw",
  optim_args = list(weight_decay = 1e-5),
  .seed = 123,
  verbose = FALSE
  )

# 4) Predict survival curves on held-out test patients
t_max <- as.numeric(stats::quantile(train$time, probs = 0.90, na.rm = TRUE))
time_grid <- seq(0.1, t_max, length.out = 100)

pred_test <- predict(mod, newdata = test, type = "survival", times = time_grid)
S_test <- extract_survmat(pred_test)

stopifnot(nrow(S_test) == nrow(test), ncol(S_test) == length(time_grid))

# 5) Cluster predicted curves with unsurv

fit_uns <- unsurv(
  S = S_test,
  times = time_grid,
  K = 3,
  K_max = 3,
  distance = "L2",
  enforce_monotone = TRUE,
  smooth_median_width = 3,
```

```r
  standardize_cols = FALSE,
  eps_jitter = 0.0,
  seed = 123
  )

cluster_rmst <- rowMeans(fit_uns$medoids)
cluster_order <- order(cluster_rmst)
relabel <- setNames(seq_along(cluster_order), cluster_order)
pred_cluster <- unname(relabel[as.character(fit_uns$clusters)])

true_test <- test$true_cluster

contingency <- table(
  True = factor(true_test, levels = 1:3),
  Predicted = factor(pred_cluster, levels = 1:3)
  )

ari <- adjusted_rand_index(true_test, pred_cluster)
accuracy <- sum(diag(contingency)) / sum(contingency)

cat("\nTrain / test sizes:\n")
print(c(train = nrow(train), test = nrow(test)))

cat("\nTest-set true clusters:\n")
print(table(true_test))

cat("\nunsurv recovered clusters on survdnn predictions:\n")
print(table(pred_cluster))

cat("\nTrue vs predicted (test set):\n")
print(contingency)

cat("\nRecovery metrics:\n")
cat(sprintf("Ordered-label accuracy: %.3f\n", accuracy))
cat(sprintf("Adjusted Rand Index: %.3f\n", ari))

# 6) Manuscript-ready figure:
#    left = all survdnn predicted curves
#    right = same curves colored by unsurv cluster + medoids
curve_df <- data.frame(
  patient_id = rep(seq_len(nrow(S_test)), each = length(time_grid)),
  time = rep(time_grid, times = nrow(S_test)),
  surv = as.vector(t(S_test)),
  pred_cluster = factor(rep(pred_cluster, each = length(time_grid)), levels = 1:3)
  )

S_med <- fit_uns$medoids[cluster_order, , drop = FALSE]
```

```
med_df <- data.frame(
  time = rep(time_grid, times = nrow(S_med)),
  surv = as.vector(t(S_med)),
  pred_cluster = factor(rep(seq_len(nrow(S_med)), each = length(time_grid)), levels = 1:3)
  )

fig_left <- ggplot(curve_df, aes(x = time, y = surv, group = patient_id)) +
  geom_line(linewidth = 0.35, alpha = 0.35, color = "grey35") +
  theme_minimal(base_size = 12) +
  labs(x = "Time", y = "Predicted survival probability") +
  theme(legend.position = "none")

fig_right <- ggplot(curve_df, aes(x = time, y = surv, group = patient_id, color = pred_cluster
  geom_line(linewidth = 0.40, alpha = 0.28) +
  geom_line(
    data = med_df,
    aes(x = time, y = surv, group = pred_cluster, color = pred_cluster),
    linewidth = 1.3,
    alpha = 1) +
  theme_minimal(base_size = 12) +
  scale_color_manual(values = c("1" = "#B22222", "2" = "#E69F00", "3" = "#1B9E77")) +
  labs(x = "Time", y = "Predicted survival probability", color = "Cluster") +
  theme(legend.position = "bottom")

# Combined with patchwork, using plain "A"/"B" corner tags (matching the
# style used for the METABRIC figures); panel content is explained in the
# figure caption rather than in an in-panel title.
p_manuscript <- fig_left + fig_right + plot_annotation(tag_levels = "A")

print(p_manuscript)

figure_path <- file.path("..", "manuscript_example_survdnn_unsurv.png")

ggplot2::ggsave(
  filename = figure_path,
  plot = p_manuscript,
  width = 11,
  height = 5,
  dpi = 300)

cat("\nSaved manuscript figure:\n")
cat(figure_path, "\n")

if (ari < 0.75) {stop("Recovery was weaker than expected; ARI fell below 0.75.")}
```
